# The effectiveness of lasers in the treatment of onychomycosis: a systematic review

**DOI:** 10.1186/1757-1146-7-34

**Published:** 2014-07-27

**Authors:** Ivan R Bristow

**Affiliations:** 1 Faculty of Health Sciences, B67, Highfield Campus, University of Southampton, Southampton SO17 1BJ, UK

**Keywords:** Laser, Onychomycosis, Nd:YAG, Fungal, Nail

## Abstract

**Background:**

Onychomycosis is a common nail pathology which has proven to be a treatment challenge to healthcare professionals. Antifungal drugs have been the mainstay of therapy for many years. Recently, laser technologies have been introduced as a treatment for onychomycosis avoiding the disadvantages of systemic and topical drug therapies, offering a rapid treatment for an often persistent nail condition. The purpose of this study was to review published evidence regarding the effectiveness of laser technologies in the treatment of onychomycosis.

**Methods:**

The primary question for this review was “what evidence is there for the use of lasers in the treatment of onychomycosis”? A systematic literature search of published papers indexed on Pubmed and Web of Science® was undertaken in June 2014 for original, published research. The primary outcome measures for efficacy were mycological cure and clearance of the affected nail (clinical cure).

**Results:**

This review returned a total of twelve eligible published studies evaluating the use of lasers in the treatment of onychomycosis. Two were randomised controlled trials, four were comparative design studies (with no placebo/control groups) and the remainder were case series. The level of evidence was generally low level reflecting predominantly small sample size and lack of control groups. The results from studies were conflicting and follow up periods for patients in studies were generally short. Many studies excluded patients with severe or dystrophic onychomycosis.

**Conclusions:**

The evidence pertaining to the effectiveness of laser treatment of onychomycosis is limited and of poor methodological quality. Future studies using a randomised controlled trial designs with larger study populations and clear procedures are required to permit a full evaluation of this emerging technology.

## Background

Lasers have been a part of podiatric practice for many years. Papers discussing their potential uses in clinical practice started to appear in the 1980s, particularly focussing on the high powered carbon dioxide (CO_2_) systems available at that time [1-3]. Much of this work discussed their ablative abilities in nail matrixectomies and their early potential for onychomycosis following total nail ablation [1] and by nail fenestration to improve topical drug delivery [4] but generally their use remained in the hands of a few specialist practitioners, mainly in the USA. This continued for several years with newer systems being slowly introduced such as the pulsed dye laser, which has been explored as a treatment for plantar warts with varying levels of success [5-8]. The expense of these early systems was prohibitive for everyday practice and so their use was limited.

In 2009, for the first time a surgical laser system was advertised in the UK Podiatry magazine *Podiatry Now* indicated for the treatment of onychomycosis. Shortly afterwards, a letter was published suggesting that this was “possibly the most radical development in the treatment of onychomycosis our profession has ever seen” [9]. Some concerns were expressed at the unproven efficacy of these new devices and the investment costs involved [10]. In addition, despite it now being nearly five years since their first introduction into practice little evidence has appeared in the professional literature demonstrating their effectiveness despite their introduction into UK clinics, evident through internet searches, offering this treatment modality. Similar concerns have been expressed in the dermatological fields with laser systems for onychomycosis being “praised uncritically and promoted at high prices” [11].

Lasers systems are attractive for the practitioner and public alike for a number of reasons. Oral drug regimens have showed effectiveness in many studies and remain the most studied intervention for this condition. However, debate is always raised about the safety of oral drugs, despite many years’ experience with these agents and safety reporting [12,13]. Antifungal drugs, like many others, are contra-indicated in patients with active or chronic liver disease [14] and are sometimes declined by patients seeking alternatives to oral medication - often to avoid potential for side-effects. Topical agents too, are considered by many to be a protracted and frequently ineffective intervention as patient compliance over the treatment period can be an issue. Lasers are often marketed as a means of improving a practice’s income and are seen as an investment which can pay for itself in a short period of time. Lasers also capture the imagination of the public as a safe, effective quick fix for a range of clinical conditions.

The proposed mechanism of action of lasers in the treatment of onychomycosis remains unclear. However, laser systems in near infra-red spectrum (780 nm – 3000 nm wavelength), which are commonly used in onychomycosis, exert their effect by direct heating of the target tissues [15]. Moreover, by using a pulsed beam instead of continuous beam, these lasers can deliver a “selective photothermolysis” [16] – delivering of a short burst of laser light energy into the target tissue causing a rapid elevation in temperature into the defined target area. Sufficient intervals between pulses can allow for tissue relaxation and cooling to occur, causing very little collateral damage to surrounding structures. In the laboratory, eradication of the common dermatophyte *Trichophyton rubrum* has been demonstrated using pulsed laser technology [17]. Studies on fungal nail clippings have demonstrated this to have a direct thermal killing effect on fungal mycelia when treatment temperatures exceed 50° centigrade [18].

Lasers for nail disease have been approved in the United States by the Food and Drug Administration (FDA). To date, devices have been approved only on their ability to temporary clear nail growth in onychomycotic nails [19], and not on definitive curative data. Consequently evaluation of their capabilities remains a necessity to inform practitioners of their effectiveness in the longer term. A literature review published in 2013 [20] which, in part, discussed laser technology in onychomycosis concluded that evidence was lacking due to small scale studies and poor design however, this was not systematic. Consequently, the author has undertaken a systematic literature review to assess the published results and evidence of effectiveness to date.

## Methods

This study was compiled adopting the “Preferred Reporting Items for Systematic Reviews and Meta-Analyses (PRISMA) guidelines” [21]. Any original study, published in a peer reviewed journal, which examined the use of a laser technology in the treatment of onychomycosis (in more than a single case) was considered for inclusion. The primary outcome measure was efficacy, only including studies which employed microbiological or histological procedures to establish an initial diagnosis of the condition, and subsequently measured the outcome either by a repeated microbiological/histological assessment or measured changes in physical nail clearance of discolouration following treatment. Studies which did not follow this procedure were excluded, as were those which stated they exclusively studied fingernail onychomycosis.

### Search strategy

**Figure 1:**
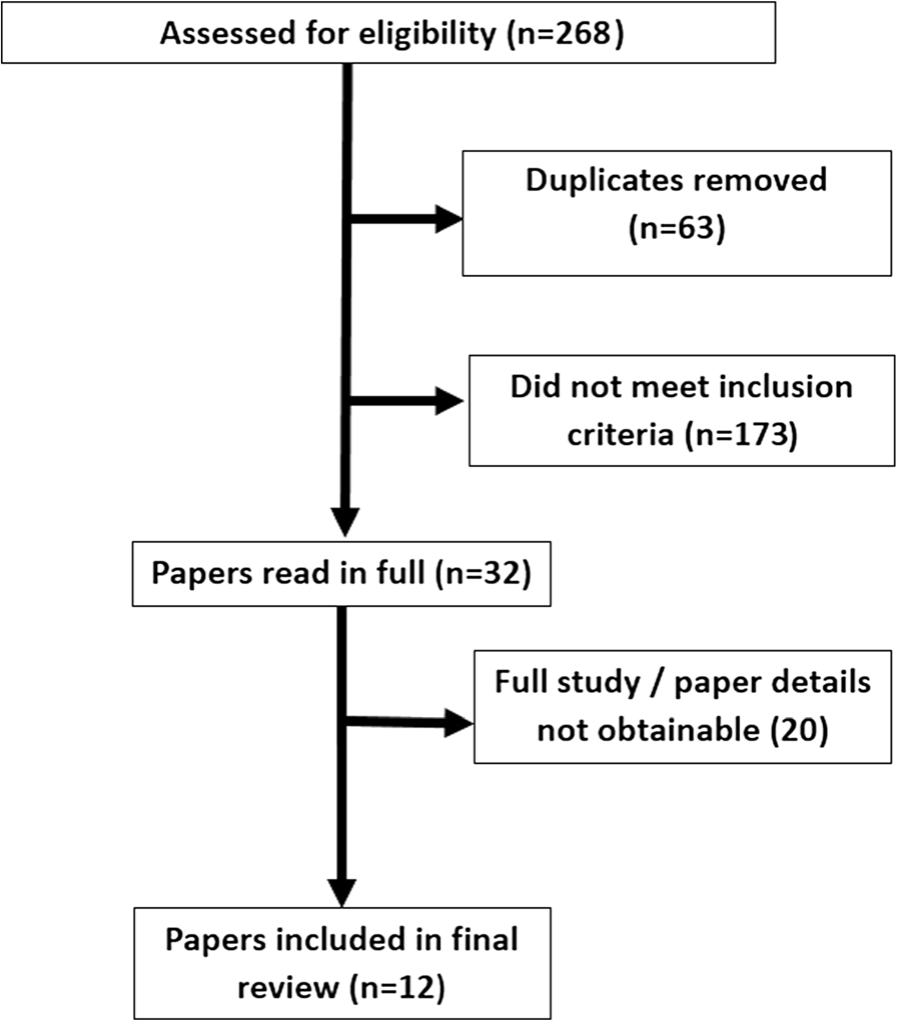
Flow Diagram of search strategy (conducted June 2014).

An electronic database search was undertaken using PubMed (US National Library of Health Database [June 2014]) and Web of Science® (June 2014) to identify papers which met the initial inclusion criteria. Searches were standardized using a combination of the keywords “onychomycosis”, “tinea unguium”, “laser”, “nail”, “rubrum”. No date limits were set, but as a relatively new modality, papers over six years old were unlikely. The initial search yielded a total of 268 potential papers. All abstracts were reviewed to remove duplicates and to remove papers which did not meet the inclusion criteria. Papers which met the inclusion parameters were then read in full by the author. Only papers written in English, with full study details available were included in the final suite of papers. This exercise was repeated twice by the author to improve the reliability of the search and prevent eligible papers being missed or excluded (Figure 1).

### Review process

Following the selection process, remaining papers were evaluated for their level of evidence using two systems as adopted by Matricciani et al. [13]. Firstly, papers were graded using the National Health and Medical Research Council (NHMRC) Hierarchy of Evidence [22] scale (see Additional file 1) and subsequently the American Occupational Therapy Association (AOTA) Evidence based literature Review Project, as adapted by Trombly and Ma [23] (see Additional file 2) as a means to identify threats to validity within the included studies. This system allowed for the objective assessment of published research, graded on the four dimensions of design sample size, internal validity and external validity.

## Results

Initial evaluation demonstrated a variety in study procedures which precluded detailed statistical comparisons and so a structured review of the papers was undertaken to examine and compare methods and results. In total, 13 papers were initially deemed eligible, however one paper [24] was an extension of an earlier published paper already included in the review [25] so was excluded.

All of the 12 remaining papers were published in the last four years [25-36]. Reflecting the novelty of this technology, four studies stated they were “preliminary” or pilot studies [26-28,31]. Two papers adopted a randomised controlled trial methodology [25,29], three were comparative designs [27,35,36] whilst the remainder were case series [26,28,30-34].

The majority of studies (10 papers) investigated the 1064 nm neodymium: yittrium-aluminum-garnet laser system (Nd:YAG) (long and short pulse types) either as a sole intervention [26-29,31,33,34,36], as a Q switched 1064 nm/532 nm wavelengths system [30], or as a comparison against a 1319 nm and broadband wavelength device [35]. One study employed an 870/930 nm dual band system [25] and another investigated the use of an ablative carbon dioxide laser as a means to fractionate nails to enhance the penetration of topical anti-fungal agents [32]. Three papers stated that fingernails had been included in the study [28,33,36] but none exclusively, so were included. A tabulated summary of all included studies is given in Table 1 along with an assessment of the level of evidence and validity measures in Table 2.

**Table 1:** Summary of studies included in the review

**Author(s)**	**Study type**	N of subjects (Nails) (Fingernails)*	**Laser**	**Diagnostic criteria**	**Max. follow up period**	**Study endpoint**
Carney et al. [26]	Case Series	10 (18)	1064 nm Nd:YAG (short Pulse). 1 treatment.	Culture	24 weeks	Decrease in area of nail visually affected measured with the Onychomycosis Severity Index (OSI) and negative cultures.
Hees et al. [37]	Comparative study	10 (20)	1064 nm (long v. short pulse) on each hallux 2 treatments over 4 weeks (side by side comparison).	Histology	36 weeks	Decrease in area of nail visually affected measured with the Onychomycosis Severity Index (OSI). Negative histology & cultures.
Hochman [28]	Case Series	8 (12) (1)*	1064 nm (short pulse). 2 or 3 treatments, 3 weeks apart.	Culture or PAS	24 weeks	Negative fungal cultures.
Hollmig et al. [29]	RCT	27 (125)	1064 Nd:YAG. 2 Treatments 2 weeks apart versus control.	Culture or PAS	52 weeks	Negative cultures and measured clearance at 3 months for all subjects, and repeated clearance measurement at 12 months for those treated with laser.
Kalokasidis et al. [30]	Case Series	131 (unknown)	1064 nm/532 Q switched Nd:YAG. 2 treatments, 30 days apart.	Microscopy & Culture	12 weeks	Decrease in area of nail visually affected measured with the Onychomycosis Severity Index (OSI) and negative cultures.
Kimura et al. [31]	Case Series	13 (37)	Nd:YAG 1064 nm (long Pulse) 2–3 treatments, 4 weeks apart.	Microscopy	*Up to* 24 weeks	Improvements in nail turbidity score and, negative culture if nail was 100% improved in turbidity score.
Landsman et al. [25]	RCT	36 (37)	870 nm/930 nm laser or sham control device. 4 treatments over 60 days.	Culture or PAS	24 weeks	Decrease in affected nail area (clearance) and negative culture or PAS stain.
Lim et al. [32]	Case Series	24 (unknown)	CO_2_ laser. 3 treatments at 4 weeks plus topical treatment.	Microscopy	24 weeks	Decrease in affected nail surface area and negative microscopy.
Moon et al. [33]	Case Series	13 (43) (12)*	1064 nm Nd:YAG (long pulse). 5 treatments at 4 week intervals.	Microscopy & Culture	24 weeks	Decrease in affected nail surface area and negative microscopy.
Noguichi et al. [34]	Case Series	12 (12)	1064 nm Nd:YAG (long Pulse) 3 treatments at 4 week intervals.	Microscopy or PCR or culture	24 weeks	Decrease in nail surface area affected and negative microscopy.
Waibel et al. [35]	Comparative study	21 (21)	1064 nm ND:YAG v. 1319 nm v. Broadband light. 4 treatments at one week apart.	Culture & PAS	24 weeks	Negative fungal culture.
Zhang et al. [36]	Comparative study	33 (154) (18)*	1064 nm Nd:YAG (long pulse) Either 4 **OR** 8 treatments - one week apart.	Microscopy & Culture	24 weeks	Negative culture and measured decrease in affected nail surface area.

**Table 2:** **Appraisal of study validity using the AOTA Evidence Based Project Scale**[23]

**Study**	NHMRC evidence level**	**Sample size**	**Internal validity**	**Threats to internal validity**	**External validity**	**Threats to external validity**
Carney et al. [26]	IV	C	2	20% attrition	b	Only DLSO mycosis of the hallux included
				Unblinded assessment		
Hees et al. [37]	III-3	C	1		b	Only T Rubrum mycosis included
Hochman [28]	IV	C	2	Antifungal cream used post intervention.	b	Type of onychomycosis treated not classified
				Variable follow up periods		
Hollmig et al. [29]	II	B	2	Blinding procedures not stated	b	Type of onychomycosis treated not classified
				18% attrition		
Kalokasidis et al. [30]	IV	A	3	Short follow up period OSI results not fully reported	b	Mainly mild/DLSO mycosis treated
				No control group		
Kimura et al. [31]	III-3	A	3	Variable no. of treatments (1–3)	b	Mainly DLSO mycosis treated
				Turbidity scoring not validated		
Landsman et al. [25]	II	B	2	Smaller control than treatment group	b	Majority cases mild to moderate mycosis
				Industry sponsorship/authorship		
Lim et al. [32]	IV	B	2	Blinding procedures for nail grading not clear.	b	Majority cases mild to moderate mycosis
						Not stated how many finger v. toe nails included
Moon et al. [33]	IV	C	2	Limited details on nail scoring system validation	b	Mainly (DLSO) included
Noguichi et al. [34]	IV	C	2	Limited detail on independence of nail score assessment	b	Severe nail disease or thick nails excluded - only mild/DLSO cases included
Waibel et al. [35]	III-3	B	2	No control/placebo group	b	Types of mycosis included not reported
				Limited detail on how nail clearance assessed and reported		
Zhang et al. [36]	III-3	B	3	No control/placebo group	b	Types of mycosis included not reported
				Limited detail on how nail clearance assessed		

**NHMRC Hierarchy of Evidence adapted from [22].

In a randomized controlled trial by Landsman and colleagues [25], 36 patients with proven onychomycosis were randomly allocated to either a laser treatment using a continuous wave Noveon™ 870 nm/930 nm laser or control sham device. Following a detailed protocol, all patients were treated at day 1, 14, 42 and day 60. Blinded assessors reviewed photographic evidence at various stages to assess and record any changes. At 6 months, 34 patients (37 toes: 26 treated and 11 controls) were eligible for analysis. Visually, only 2 treated nails had completely or markedly improved (versus 2 controls) whilst slight to moderate improvement was seen in 18 treated nails versus 3 control nails and 6 treated nails were unchanged along with 6 control nails. The study was declared as being funded exclusively by a laser manufacturer and employees of the company were listed as co-authors of the paper.

In another randomized controlled trial, Hollmig et al. [29] enrolled 27 patients with culture or PAS stain confirmed onychomycosis to receive either 2 treatments with a 1064 nm Nd:YAG laser (two weeks apart) or no treatment. At three months all patients affected nails were re-assessed by culture and measured nail clearance with an additional measurement for the treated group at month 12. The results showed that at 3 months, 33% of the laser treated group achieved a negative culture versus 20% in the control group and had more proximal nail clearance at this time, although there was no statistically significant difference between the two groups. At month 12, there was no difference in measured nail clearance between the treated and control group. The authors suggested that the laser may only have a temporary effect in onychomycosis.

Hochman [28] undertook a study of 8 patients with culture or PAS stain confirmed onychomycosis and treated them using a LightPod Neo^TM^ 1064 nm short pulsed Nd:YAG laser (Nd:YAG laser). After 2–3 treatments, three weeks apart, they were re-assessed at an unspecified follow-up time of at least 4 months. The author reported a 7 out of 8 patients showing visual improvement (although this was not quantified) and negative fungal cultures. However, in this study, no description was given about the level of nail involvement at the beginning of the study to ascertain the extent of the disease. In addition, patients were encouraged to use daily antifungal agents during treatment applied to the nail. Small numbers were used in this study and the follow up time was not formalized ranging from 16 to over 24 weeks.

Published in 2012, Kimura et al. [31] undertook a study of 13 subjects (37 toe nails) and investigated the effectiveness of the Cutera™ Nd:YAG short pulse laser in the treatment of patients with dystrophic nails, microscopically confirmed as onychomycosis. Nails were treated two or three times 4–8 weeks apart. The main outcomes assessed were evidence of clear nail growth (using a nail turbidity score) and a negative fungal clipping. The majority of patients presented with distal lateral sub-ungual onychomycosis (n = 9). At the end of the study (week 16) 19 nails (51%) showed complete clearance (clear nail and negative microscopy) with 30 nails (81%) showing from moderate to complete improvement. The authors declared that the equipment for the study had been loaned from the manufacturers but did not state if the results were independent of the company.

In a more recent study by Moon et al. [38] 43 toenails and 12 finger nails with culture and PAS stain confirmed onychomycosis underwent 5 treatments using a Nd:YAG 1064 nm laser system at four week intervals. At 24 weeks from the start of the study nails were assessed for surface clearance and negative cultures. One month after the final treatment 30 of the 43 nails had negative microscopy. Four nails achieved a complete cure (negative microscopy and complete visual clearance in the nail plate). Eight patients were reported to have achieved >80% nail clearance and 31 nails (50-80% clearance of nail surface area).

Using the Pinpointe™ Laser System (1064 nm Nd:YAG) with a long pulse duration, Zhang and colleagues [36] randomly assigned into two treatment groups, 33 microscopically and fungal culture positive patients (154 nails) with onychomycosis to either 8 treatments at one week intervals (group 1) or 4 treatments at one week intervals (group 2). Patients were followed up for 24 weeks. There was no significant difference in the mycological cure rates which were 51% (group 1) and 53% (group 2) at 24 weeks. Interestingly, they reported recurrence on the disease in 10 nails (5 patients) within a 2–4 month period after the study, suggesting that the laser had only temporarily inhibited growth and not destroyed the fungus outright.

Waibel et al. [35] undertook a study using three types of laser light (1064 nm, 1319 nm and broadband filtered flash light) randomly assigning 21 patients to one of the modalities. All patients had PAS confirmed disease and had positive microscopy cultures. Each nail was treated with 10 minutes of laser light and received four, weekly treatments and were assessed at 1, 3 and 6 months. Tissue temperature was also recorded in this study to suggest what effect the laser was having on the treated area. The authors reported improvement in the nail appearance with “clearing” and a high satisfaction rate, with only mild discomfort reported by patients. Although these findings were not quantified, they reported 100% negative cultures for the 1064 nm laser system and Broadband light with one failure for the 1319 nm system. The sample sizes for this study were small with 7 subjects in each arm. A measured temperature of 46 degrees centigrade was achieved for all treatments. Based on the results the authors concluded that this was a lethal temperature which would achieve the desired outcome.

In a larger study Kalokasidis et al. [30] treated 131 patients with microbiologically confirmed onychomycosis using a Q-Clear™ Q-Switched Nd:YAG 1064 nm/532 nm laser following nail reduction with a drill. Patients underwent two treatments, 30 days apart, using both wavelengths each time and then were reviewed at two months. The results demonstrated an impressively high cure rate, assessed by microscopy and culture, of 95.4% in the study group which is much higher than other studies have reported. The data suggested that distal sub-ungual onychomycosis and superficial white onychomycosis are very amenable to this modality whilst lesser results were observed in patients with severely dystrophic nails. As the authors state the follow up time for assessment in this study was very short as effective nail growth can take up to 12–18 months to fully reveal the nail post-operatively. Although the nail severity index was used initially to assess nail involvement, subsequent score post-intervention were not fully reported.

In a Japanese paper, Noguchi et al. [34] treated the hallux nails of 12 mycological positive patients with a GentleYAG™ 1064 Nd:YAG laser. Nails with severe disease were excluded (>75% surface area affected or >3 mm plate thickness) so the treated group only presented with distal lateral sub-ungual onychomycosis type. All patients underwent the minimum of 3 treatments at 4-week intervals with nail turbidity (clearance) assessed at 3 and 6 months. Mycological cure rates were not assessed in this study, only visible improvement measured through changes in affected nail surface area. Only three patients showed a significant improvement (25%) with two showing improvement (16.7%) and six patients (50%) showing no improvement or worsening. The authors concluded from their results that this procedure was no better than topical nail lacquer therapy based on the cure rates achieved.

Carney et al. [26] as part of a range of experiments undertook a study of 10 patients (18 nails) with onychomycosis. In the clinical part of their study, they selected 10 patients with mycologically proven onychomycosis and undertook a treatment using a single 1064 nm Nd:YAG system. This study included rigorous assessment of the nails before and after using the OSI measure as well as the mycological evidence to assess outcomes with a 24 week programme. They could not show significant improvements in mycological or clinical cure rates using this laser system and regime.

Hees et al. [27] reported a ten patient pilot study also employing a comparative design of the two types of 1064 Nd:YAG systems (short pulse versus long pulse). Patients included had all grades of onychomycosis caused by T rubrum with mycological confirmation in both halluces and underwent a left hallux/right hallux comparison of the two laser systems with a two treatment regime spaced four weeks apart to both nails. Nail changes were independently assessed by two observers using standardised photography and the OSI [39] with a nine-month follow up. Despite mycological clearance rates of around 65% in this small sample, the OSI grading changed very little for participants – improving slightly within the first six months and then reversing slightly at the end of the nine month study. In conclusion, although 65% had mycologically been cured, clinically only 4 cases had shown visible improvement.

Only one study evaluated the carbon dioxide ablative laser (CO_2_) in the treatment of onychomycosis [32]. The function of laser therapy in this study was to render the nail more permeable to facilitate penetration of concurrent topical amorolfine to affected nails which was then evaluated. After three treatments at 4 week intervals, 24 patients with confirmed onychomycosis were assessed at six months for negative cultures and improvements in clear nail growth. At this time, 50% of patients had negative culture and had 100% nail clearance whilst only 2 patients (8%) showed no response. As the authors point out, mild forms of onychomycosis such as superficial white onychomycosis responded best whilst no improvement was seen in the totally dystrophic cases.

## Discussion

This review of published papers has yielded 12 studies investigating the use of lasers in the treatment of onychomycosis. Sample sizes in all studies were generally small ranging from 8 [28] to 131 patients [30], with six studies having 20 or fewer patients [26-28,31,33,34]. Only one paper offered a detailed design and protocol with a control (sham) intervention [24].

On review of the data available, a number of issues arise. Firstly, looking at early results it is clear that there is no consensus on laser effectiveness with conflicting study results being evident. This, in part, is due to the heterogeneity of the study designs at many stages. Although all papers reviewed onychomycosis in adults, selection criteria showed variation. Most focussed on older adult samples reflecting the fact that onychomycosis is a disease are more prevalent in this age group [40] however, definitive diagnosis for inclusion relied on a single test result in some studies [25-29,31,32] or a combination of tests such as Periodic Acid-Schiff (PAS) stain, microscopy, positive fungal culture or polymerase Chain Reaction (PCR) in others [30,33,35,36,41]. The variability of these tests have been investigated. Weinberg et al. [42] examined 94 nail samples and suggested the sensitivities of KOH as 80%, PAS 92% and culture 59%. More recently developed PCR techniques are considered to the new standard technique in detecting dermatophyte presence [43] with detection rates similar to PAS staining technique [44].

The research to date highlights the difficulty in what constitutes an effective “cure” in onychomycosis and how it is measured. Mycological cure is defined as clearance of the nail based on negative mycological test findings such as microscopy, culture and PAS staining, however this can be complicated when an individual may show mixed results when exposed to a range of different mycological tests. Moreover, a negative culture result may not equate to improvement in the nail appearance (known as “clinical cure”). A “complete cure” is a combination of mycological and clinical cure – effectively the nail is free from fungus and visually, returns to normal.

Visual appearance in some studies was based on patient satisfaction levels [36] whilst others measured clear nail emergence [25,31,34] or formalised the changes in the amount of nail plate surface affected and its associated changes. The OSI [39] was a commonly used instrument [26,27,30] and categorised nails based on the amount of nail surface area affected. Other studies made no formal visual assessment [28,35]. As previous papers have discussed [45,46] assessing what constitutes a cure is difficult, requiring further work to provide a meaningful outcome for the clinician and the patient.

Another factor for consideration is the duration of the study. Most studies ran between 12–24 weeks [25,26,28,30-36], with one recent study continuing to 36 weeks [27] and one to 12 months [29]. In fully assessing the effectiveness of laser therapy, it is perhaps important to consider nail growth rate. In adults, toenails grow around 1.0 mm/month however in the elderly, the rate of nail growth decreases by approximately 0.5% per year between 25 to 100 years of age [47]. In addition, it has been shown that nails infected with dermatophytes grow at a slower rate than uninfected nail plates, proportional to amount of nail affected [48]. Consequently, for a full nail growth to occur post-intervention, a longer time period of up to 24 months maybe a more suitable way to assess effectiveness. The Hees et al. study [27], which suggested deterioration in the visual appearance of the some nails, had a longer follow up than most studies whilst Zhang and colleagues [36] reported rapid relapse in five patients (a total of 10 nails). Moreover, Hollmig et al. [29] noted that although modest improvement in nail clearance was observed in their laser treated group at 3 months compared to the control group, this was not sustained at 12 months, suggesting only a temporary effect.

To compound the issue, as studies become longer in duration the risks of relapse or reinfection potentially increase. Relapse is defined as a recurrence of the nail infection, resulting from insufficient clearing of the original infection from the nail, whereas reinfection is a new infection occurring in a nail that has been previously cleared of all infection. It has been shown that reinfection is a common occurrence in onychomycosis [49,50] probably occurring as the patient re-acquires dermatophytic fomites from previously worn footwear and hosiery. In such studies, the use of preventative measures such as shoe and sock disinfection or topical nail lacquers and antifungal creams may be a useful addition following completion of laser treatment to counteract the effect of reinfection. Only two studies in this review employed the use of a topical antifungal applied to the skin or nails after treatment deliberately to reduce reinfection [25,28].

Other issues worthy of discussion, include nail thickness and severity of the infection. Increased nail thickness in any type of treatment for onychomycosis acts as a potential barrier. Noguichi et al. [34] measured nail thickness and excluded those with a nail thickness of greater than 3 mm suggesting that the 1064 nm laser can only penetrate down to this depth. Kalokasidis et al. [30] employed the use of a nail drill prior to treatment in their study, Hees et al. stated that they did not in their study deliberately seek to drill nails prior to therapy [27]. All other studies did not state, either way, if nail reduction was employed [25,26,28,29,31-33,35,36]. The use of nail drilling prior to antifungal drug use has been shown to be effective in improving cure rates [51,52] but its effect in laser treatment remains a point to be tested.

The type of onychomycosis is a factor which may the affect the outcome of treatment. The four main types are distal lateral sub-ungual onychomycosis (DLSO), superficial white onychomycosis (SWO), proximal white sub-ungual onychomycosis (PWSO) and total dystrophic onychomycosis (TDO). The extent of the nail infection will potentially have a bearing on the treatment success. The DLSO and PWSO varieties showed in some studies being more responsive to treatment possibly owing to their mild to moderate presentation when compared to the more severe proximal PSO and TDO varieties. Clear documentation of the profile of type of onychomycosis presenting in study cohorts would permit a clearer judgement on the lasers effectiveness. Four studies [28,29,35,36] did not profile the onychomycosis at presentation whilst two only included DLSO or SWO [26,34]. The remaining five studies included all types [25,27,30-32], however in four of these studies, the vast majority were DLSO cases [25,30-32].

Setting up and running clinical trials for new laser technologies can be costly and time consuming. To that end, sponsorship from industry may not be entirely objectionable in researching new devices [11] but it is an important factor to be considered in judging the validity of any study. Of the 11 included studies, only one paper made no declaration of competing interests [36] whilst four papers declared competing interests ranging from loan of equipment [31], to individual author involvements with associated companies [26,35], through to full sponsorship and involvement in study design and authorship in one study [25].

This paper set out to systematically review current evidence in the treatment of onychomycosis employing laser technologies. However, there are potential limitations to this work. Firstly, whilst this review includes papers published and indexed on two large databases (Web of Science® and PubMed), it must be stated that these libraries are not exhaustive and therefore cannot cover all relevant publications. As an emerging modality, searches of the internet reveal much more literature in the form of documents, papers and posters but it is difficult to objectively ascertain their origins, peer review status or whether any conflicts of interest exist. Therefore, only recognised databases have been used for this review. In addition, only studies written in English and which are readily accessible through normal methods have been included.

## Conclusion

In the last five years, papers have been published investigating the application of lasers in treating onychomycosis. Twelve studies were identified and included in this review. Most of the published data to date is reported at a low level of evidence predominantly case series involving small numbers of patients and with only two small randomized controlled studies. Outcomes appeared to be measured on visual nail clearance measurements following treatment, mycological evidence of cure or both. Studies with longer follow up periods suggest nail infection relapse occurring in those treated with laser which warrants further investigation. However, overall conflicting results are shown in this review of studies with no clear evidence of efficacy. There were no studies published comparing laser with more traditional therapies in the treatment of onychomycosis. Comparative studies are required with improved design such as longer follow up periods, classification of the type of nail infection and control interventions to truly assess the effectiveness of laser devices in the management of onychomycosis.

## Abbreviations

AOTA: American Occupational Therapy Association; DLSO: Distal lateral sub-ungual onychomycosis; FDA: Food and Drug Administration; Nd:YAG: Neodymium:Yttrium Aluminum Garnet (laser); OSI: Onychomycosis severity index; PAS: Periodic Acid-Schiff stain; PCR: Polymerase Chain Reaction; PWSO: Proximal white sub-ungual onychomycosis; RCT: Randomised Controlled Trial; SWO: Superficial white onychomycosis; TDO: Total dystrophic onychomycosis.

## Competing interests

The author declares he has no competing interests.

## Authors’ contributions

IB was responsible for the drafting of the paper and conducting the review. All authors read and approved the final manuscript.

## Supplementary Material

Additional file 1 **NHMRC Hierarchy of Evidence **[22]**.** Click here for file

Additional file 2 **Levels of evidence for the AOTA Evidence-Based Practice Project **[23]**.** Click here for file
