# Supplementary material for: The effectiveness of lasers in the treatment of onychomycosis: a systematic review
Source: J Foot Ankle Res. 2014 Jul 27;7:34. doi: 10.1186/1757-1146-7-34 (PMC4124774; doi:10.1186/1757-1146-7-34)
Supplement: Additional file 1 — NHMRC Hierarchy of Evidence [22]. [file 1757-1146-7-34-S1.docx]

| **Level of Evidence** | **Study Design** |
| --- | --- |
| I* | A systematic review of level II studies |
| II | A randomised controlled trial |
| III-1 | A pseudo-randomised controlled trial |
| III-2 | A comparative study with concurrent controls |
| III-3 | A comparative study without concurrent controls |
| IV | Case series with either post-test or pre-test/post test outcomes |
