# Supplementary material for: The effectiveness of lasers in the treatment of onychomycosis: a systematic review
Source: J Foot Ankle Res. 2014 Jul 27;7:34. doi: 10.1186/1757-1146-7-34 (PMC4124774; doi:10.1186/1757-1146-7-34)
Supplement: Additional file 2 — Levels of evidence for the AOTA Evidence-Based Practice Project [23]. [file 1757-1146-7-34-S2.docx]

| Level | Definition |
| --- | --- |
| Design | |
| I | Randomised trial: Comparison of two or more groups or conditions in an experiment with random assignment to group or sequence of conditions in a repeated-measures design. |
| II | Non-RCT: Comparison of two or more groups or treatments in a quasi-experiment without randomisation to group, condition or sequence. |
| III | Non-RCT: Comparison of one group pre and post treatment. |
| IV | Single subject design: One subject measured at intervals throughout an intervention. |
| V | Narratives and case studies. |
| Sample Size | |
| A | n ≥ 50 persons per condition or group or observations in a single-subject design. |
| B | n ≥ 20 persons per condition or group or observations in a single-subject design. |
| C | n ≤ 20 persons per condition or group or observations in a single-subject design. |
| Internal Validity | |
| 1 | High internal validity: No strong alternative explanation for outcome or other threats to validity. Such as attrition, unblended evaluation, unequal treatment, or spontaneous recovery. |
| 2 | Moderate internal validity: No strong alternative explanation for outcome but one or two threats to validity exist. |
| 3 | Low internal validity: Does not meet criteria for 1 or 2. |
| External Validity | |
| a | High external validity: Participants represent population *and* treatment represents current practice *or* has strong theoretical support *and* the research was done in a natural (clinic or home) setting. |
| b | Moderate external validity: Has two of the criteria listed for a. |
| c | Low external validity: Has one or fewer criteria listed for a. |
